# Obesity in young adults: The differentiated impact of *LEP*, *LEPR*, and *FTO* gene variants

**DOI:** 10.1590/1678-4685-GMB-2025-0265

**Published:** 2026-07-03

**Authors:** Leandro da Rocha Lima, Roberta Luisa Barbosa Leal, Marcos Vinícius Guimarães Soares, Gabriela Eduardo França de Araujo, Ana Beatriz Martins Topciu Fonseca, Alessandra dos Santos Ribeiro, Beatriz de Abreu Lopes de Azevedo, Marcelo Ribeiro-Alves, Kenia Balbi El-Jaick

**Affiliations:** 1 Universidade Federal do Estado do Rio de Janeiro, Programa de Pós-Graduação em Biologia Molecular e Celular, Rio de Janeiro, RJ, Brazil.; 2 Universidade Federal do Estado do Rio de Janeiro, Escola de Nutrição, Rio de Janeiro, RJ, Brazil.; 3 Fundação Oswaldo Cruz, Instituto Nacional de Infectologia Evandro Chagas, Laboratório de Pesquisa Clínica em IST-AIDS, Rio de Janeiro, RJ, Brazil.; 4 Universidade Federal do Estado do Rio de Janeiro, Instituto Biomédico, Departamento de Genética e Biologia Molecular, Rio de Janeiro, RJ, Brazil.

**Keywords:** Early-onset obesity, genetic predisposition to disease, genetic polymorphism, leptin

## Abstract

Obesity is a global public health issue, increasingly affecting young adults. Its association with other diseases highlights the urgency of developing prevention strategies. Genetic factors play a significant role in susceptibility to obesity, making the identification of risk-associated variants essential for prevention strategies. Therefore, this study aimed to analyze *LEP*, *LEPR*, and *FTO* variants as potential genetic risk factors for obesity in Brazilians aged 18-35 years. The participants were classified with, or without obesity/overweight. Genotyping was performed by ASO-PCR, RFLP, and DNA sequencing. A questionnaire was applied to collect anthropometric data, and personal and family medical history. Preliminary analyses indicated that obesity was significantly associated with individuals over 25 years of age; therefore, to specifically investigate early-onset obesity, the primary genetic association analyses were restricted to the 18-25 age group. A significant association was found between the *LEP* rs7799039 variant and BMI ≥ 25 Kg/m², and *LEP* rs17151919 was strongly associated with BMI ≥ 30 Kg/m² in this age group. These findings underscore the importance of identifying genetic variants that increase the risk of obesity in young adults and suggest contributing to the development of more effective and personalized prevention strategies, integrating knowledge from genetics, medicine, and nutrition.

## Introduction

Polymorphisms in *Leptin* (*LEP*, HGNC:6553), *Leptin receptor* (*LEPR*, HGNC:6554) and *FTO alpha-ketoglutarate dependent dioxygenase* (*FTO*, HGNC:24678) genes have been associated with obesity in different populations (Legry et al., 2009; Carlos et al., 2013; de Oliveira et al., 2013; da Dasgupta et al., 2015; Fonseca *et al*., 2019; Yaghootkar et al., 2020; Piwonska et al., 2022; Saqlain et al., 2022; Park and Choi, 2023). However, studies of some of these variants have often presented divergent results, mainly regarding the association of *LEP* variants: rs2167270 and rs7799039, and *LEPR* variants: rs1137101, rs1137100, and rs1805094 with the body mass index (BMI), and serum leptin levels (de Oliveira *et al*., 2013; Dasgupta *et al*., 2015; Duan et al., 2020; Garavito et al., 2020; Ponasenko et al., 2022; Saqlain *et al*., 2022; El Fessikh et al., 2023; Park and Choi, 2023). In contrast, an *in silico* analysis has suggested that *LEPR* variants may compromise leptin receptor function, a finding that corroborates the positive associations observed between obesity and these variants (El Fessikh *et al*., 2023). 

In contrast, studies of *FTO* variants suggest that they have a more robust association with increased risk for the development of obesity, even though their effects have been recognized as indirect, since *FTO* intronic variants were associated with the higher expression of the *IRX3* (Iroquois-class homeobox 3) gene. This gene is expressed in hypothalamic pro-opiomelanocortin neurons (POMC), and changes in their expression levels affect body adiposity, and energy expenditure (Smemo et al., 2014; Schneeberger, 2019). 

It is well known that common multifactorial obesity is associated with several genetic, epigenetic, and environmental risk factors (Diels et al., 2020). These diverse causal variables make obesity difficult to treat in a unique way and require a comprehensive analysis to understand the proportion of genetics and environmental effects and their individual impacts, in order to develop better strategies for its prevention and treatment. In this context, the various gene-nutrient interactions show that personalized nutrition is also a fundamental component of precision health (Kiani et al., 2022).

Another current concern regarding obesity is its increasing prevalence among young adults in recent years. In the Brazilian population aged 18 to 24, the prevalence of obesity showed a significant increase, from 9% in 2022 to 17.1% in 2023 (COVITEL, 2023). High prevalence of obesity in young adults has also been reported in other populations. In 2022, a study in the English population revealed that 11% of men and 17% of women aged 16 to 24 years presented obesity (NHS, 2024), while a study in the US population reported that 27.1% of men and 33.3% of women aged 20 to 24 presented obesity, in 2021 (Ng et al., 2024). These numbers are alarming, and estimates indicate that they will continue to grow among individuals up to 36 years of age in the coming years (De Pauw et al., 2022). Studies conducted with England, USA and Austria populations have indicated that the weight gain is greatest in the 20s and begins to decrease over 10 or 15 years (Peter et al., 2014; Dutton et al., 2016; Katsoulis et al., 2021). In this context, it is important to highlight that weight gain from early adulthood to middle age may be associated with higher risks of serious chronic diseases and a lower probability of healthy aging, reinforcing the importance of prevention and early treatment of obesity in younger individuals (Zheng et al., 2017). Thus, for a more accurate analysis, it is essential to study groups by specific age range, considering the context and the condition to be studied (Geifman et al., 2013).

Therefore, the objective of this work was to investigate the distinct contribution of variants in the *LEP*, *LEPR*, and *FTO* genes to the risk of obesity in young adults, considering the particularities of this group, such as lifestyle habits, and physiological and behavioral characteristics, which differentiate them from other age groups. In addition, the study also searches to identify additional risk factors such as personal and family history of health problems, that could contribute to predicting the increased risk of weight gain in early adulthood.

## Subjects and Methods

### Study design, sample, and data collection

This study was designed as an observational case-control study. Biological samples of peripheral blood or saliva were collected from volunteers aged 18 to 35 years, mostly students from Federal University of the State of Rio de Janeiro (UNIRIO), residing in Rio de Janeiro, from 2018 to 2023. The exclusion factors for participation in the study were: i) not having Brazilian nationality; ii) age under 18 or over 35 years; iii) pregnancy; iv) known genetic syndrome. The individuals were divided into two groups according to their body mass index (BMI). For participants aged 18 to 19 years, BMI was converted to a Z-score using the World Health Organization (WHO) growth reference data for 5-19 years (de Onis et al., 2007) to account for age and sex differences. Classification was as follows: “with obesity” (BMI ≥ 30 kg/m², or BMI Z-score ≥ 2) and “without obesity” (BMI < 30 kg/m², or BMI Z-score < 2). Also, additional analyses included the groups: “with overweight or obesity” (BMI ≥ 25 kg/m², or BMI Z-score from 1 to 1.99) and “without overweight or obesity” (BMI < 25 kg/m², or BMI Z-score < 1). The BMI and BMI Z-score of the individuals was classified according to World Health Organization (WHO).

Anthropometric data (weight and height) were directly measured in 54% of the volunteers (n=95), confirming their self-reported measurements by filling out a questionnaire, while the remaining 46% (n=81) provided self-reported data. For the self-reported group, the validity of the data was supported by the strong correlation observed in the measured subgroup (r > 0.90), consistent with previous validation studies (Gorber et al., 2007; Fayyaz et al., 2024). However, some participants reported significant weight loss through diet and/or medical treatments prior to their participation in the study. Consequently, the highest self-reported weight achieved by the participants within the study age range (18-35 years) was utilized for association analyses. This approach aimed to minimize the potential misclassification of genetically predisposed individuals who may have undergone significant weight loss prior to enrollment. The current weight of 18-year-old participants was considered to calculate their highest BMI, since the highest BMI values reached before the age of 18 years were not considered in the study. 

In addition to demographic and anthropometric information, a structured questionnaire was administered to all 176 eligible participants. The questionnaire collected data on history of childhood overweight/obesity and perception of satiety, which was assessed by the question “How would you define your satiety after a standard meal?” (categorized as “Long satiety”, “Short satiety”, or “Low satiety”). Participants also self-reported whether they or their direct family members (parents, siblings, and grandparents) had received a medical diagnosis of vitamin D deficiency, anxiety, depression, thyroid disorder, hypertension, hypercholesterolemia, hyperinsulinemia, and type 2 diabetes mellitus. 

### Molecular analyses

DNA from blood and saliva samples was extracted by commercial kits, following the manufacturers protocol.

The identification of the variants *LEP* rs2167270, *LEP* rs17151914, *LEP* rs17151919, *LEPR* rs1137100, *LEPR* rs1805094, and *FTO* rs9939609 was performed by Polymerase Chain Reaction with allele-specific oligonucleotide (ASO-PCR), and *LEP* rs7799039 and *LEPR* rs1137101 variants were identified using Restriction Fragment Length Polymorphism (RFLP), with the endonucleases FastDigest HhaI and FastDigest MspI (Thermo Scientific, MA, USA), respectively. Target sequences were amplified by PCR using primers designed for this study (Table 1). ASO-PCR test’s standardization was done with DNA samples of genotypes already known by sequencing. After all samples genotyping, part of the results was also confirmed by sequencing at the Technological Platforms Network of the Oswaldo Cruz Foundation (FIOCRUZ), revealing 100% test sensitivity and specificity.

The PCR protocols followed the recommendations of GoTaq^®^ G2 Hot Start Colorless Master Mix (Promega, WI, USA) and Go Taq^®^ G2 Hot Start Polymerase (Promega, WI, USA) respective manufacturers, using 0.4 μM of each primer, in 25 μL total volume.

**Table 1-:** Primers used for ASO-PCR and RFLP genotyping of *LEP*, *LEPR*, and *FTO* variants.

Variant	Primer Strand	Primer Sequence 5’->3’	Tm	Amplicon
rs7799039 (*LEP*)	Forward	GAAGCGATGGATGCACAGTTG		
	Reverse	TCCAGCCGATCTCTCTGTTC	64 °C	401 bp
rs2167270 (*LEP*)	Forward	TGGTCCTTGCGCCATAGTCG		
	ASO Wild Type Reverse	GGGCCTTACCTTGCAACCGC	74 °C	342 bp
	ASO Mutant Forward	CGTAGGAATCGCAGCGCCAA	68 °C	381 bp
	Reverse	GGTGGGACATCCTGATCTTTGG	68 °C	684 bp
rs17151919 (*LEP*)	Forward	AGGAATCTCGGAGACCAGCTTAG	64 °C	855 bp
	ASO Mutant Forward	CCAGTATGCCTTCCAGAAACA	64 °C	544 bp
	ASO Wild Type Forward	CCAGTATGCCTTCCAGAAACG	65 °C	544 bp
	Reverse	GGACAAGACAACTCTTGTTGATGG		
rs17151914 (*LEP*)	Forward	AGGAATCTCGGAGACCAGCTTAG		
	Reverse	GGACAAGACAACTCTTGTTGATGG	64 °C	855 bp
	ASO Mutant Reverse	TCAGCATGTGGGAGGAATCA	64 °C	166 bp
	ASO Wild Type Reverse	TCAGCATGTGGGAGGAATCG	65 °C	166 bp
rs1137100 (*LEPR*)	Forward	GAGCACTACATGGTTTAATCTCA		
	Reverse	GCCATAAGACATCTATTTCATACAG	64 °C	503 bp
	ASO Mutant Reverse	AGAATTTACTGTTGAAACAAATGTCC	64 °C	403 bp
	ASO Wild Type Reverse	AGAATTTACTGTTGAAACAAATGTCT	65 °C	403 bp
rs1137101 (*LEPR*)	Forward	AGCCTATCCAGTATTTTCATATCTG		
	Reverse	CATCTACCATCATTACAGTGTTAAGC	64 °C	391 bp
rs1805094 (*LEPR*)	Forward	CTGAAGGCAGAGAACACAGAATCAG		
	Reverse	CTAACACTATGCCAGTGCTTCAG	61 °C	668 bp
	ASO Mutant Reverse	CCAAAGTAAAGTGACATTTTTCTCG	65 °C	482 bp
	ASO Wild Type Reverse	CCAAAGTAAAGTGACATTTTTCTCC	61 °C	482 bp
rs9939609 (*FTO*)	Forward	GGCTCTTGAATGAAATAGGATTC	60 °C	303 bp
	ASO Mutant Forward	CTTGCGACTGCTGTGAATTTAG	66 °C	176 bp
	ASO Wild Type Forward	CTTGCGACTGCTGTGAATTTTG	66 °C	176 bp
	Reverse	ATGTCCAAACAGTAGGTCAGG		

### Statistical analysis

In the descriptive analyses in relation to the usual BMI categories, Mann-Whitney U tests were used to compare continuous numerical variables, and Chi-square tests were used for categorical and clinical demographic variables. In the genetic association analyses, the genotypic and allelic frequencies of each variant were determined by direct counting, and the chi-square test was used to evaluate the deviation from the Hardy-Weinberg equilibrium. The homozygous genotypes of the highest frequency allele in our sample were compared with the other genotypes, including the allele with the lowest frequency (carriers), to better observe the differences caused by the variation. The protection/risk estimate is presented as adjusted odds ratios (aORs) with 95% confidence intervals (CI95%) for each variant and estimated using unconditional logistic regression models. The haplotype frequencies were estimated by maximum likelihood, and the phase uncertainty was included in statistical models applied for association analyses. The most frequent haplotypes of the observed markers of *LEP*, *LEPR*, and *FTO* genes were considered references for the haplotype analyses. Phenotypic traits, e.g., Sex, Race, and Age, were included as confounding factors in the modeling of all other genetic analyses to eliminate possible sampling biases. The analyses were performed in the R software, version 4.1.2, with the libraries ‘genetics’ and its dependencies. A post-hoc power analysis was conducted using the G*Power software (Faul et al., 2007) to validate the study’s robustness.

The research was approved by the Research Ethics Committee - UNIRIO - CEP / HUGG (CAAE: 55438116.1.0000.5258) in accordance with ethical standards, ensuring the well-being of the participants. Informed consent was obtained from all the participants.

## Results

Because of the age limit, only 176 of the 206 volunteers were eligible to the study. Demographic and BMI analyses revealed that the majority of participants were women, self-identified as White, between 19 and 25 years of age, and presented a BMI below 29.9 kg/m² (Table 2).

**Table 2 -:** Summary of the studied population characteristics (n = 176).

Feature	Total		Without obesity		With obesity		p-value
	n	%	n	%	n	%	
Sex/Gender							
Female	120	68.2	96	54.6	24	13.6	0.284
Male	56	31.8	40	22.7	16	9.1	
Race/Ethnicity							
White	107	60.8	84	47.7	23	13.1	0.107
Brown	44	25.0	31	17.6	13	7.4	
Black	24	13.6	21	11.9	3	1.7	
Other	1	0.6	0	0	1	0.6	
Age							
18 years	8	4.5	6	3.4	2	1.1	0.008
19-25 years	132	75.0	109	61.9	23	13.1	
26-35 years	36	20.5	21	11.9	15	8.5	

The association analysis between age and obesity revealed that the age group between 26 and 35 years were significantly associated with BMI ≥ 30 kg/m² (aOR: 3.4; 95% CI 1.52-7.62; p = 0.005), and also with BMI ≥ 25 kg/m² (aOR: 2.76; 95% CI 1.23-6.2; p = 0.027) (Table 3). Thus, the association analysis with overweight and obesity was conducted considering only the individuals who achieved their highest weight throughout life when aged 18 to 25 years (n= 140), aiming to observe whether these individuals would present a distinct impact of genetic factors on the development of early obesity, since this group was at a lower time exposure to otherwise risk factors. Additional analyses, including the satiety perception and medical history of participants, were also performed considering individuals who self-declared their maximum weight between 18 and 25 years. Table 4 presents the characterization of the sample, considering the Body Mass Index classification of the participants (with or without obesity). No participants reported having been diagnosed with type 2 diabetes, and only one participant with overweight declared having been diagnosed with hyperinsulinemia.

**Table 3 -:** Association analysis between age and BMI ≥ 30 kg/m² (obesity), and BMI ≥ 25 kg/m² (overweight or obesity).

Obesity (BMI ≥ 30 kg/m²)								
Age	Total		Without obesity		With obesity		aOR (95% CI)	p-value
	n	%	n	%	n	%		
19-25	132	75	109	80.2	23	57.5	Reference	
0-18	8	4.5	6	4.4	2	5.0	1.64 (0.31-8.74)	0.560
26-35	36	20.5	21	15.4	15	37.5	**3.4 (1.52-7.62)**	**0.005**
Overweight/Obesity (BMI ≥ 25 kg/m²)								
Age	Total		Without overweight/obesity		With overweight/obesity		aOR (95% CI)	p-value
	n	%	n	%	n	%		
19-25	132	75	68	82.9	64	68.09	Reference	
0-18	8	4.5	4	4.9	4	4.26	1.09 (0.26-4.55)	0.909
26-35	36	20.5	10	12.2	26	27.66	**2.76 (1.23-6.2)**	**0.027**

aOR: adjusted Odds Ratio; CI: Confidence Interval. Adjustments were made for Sex and Race. Reference groups are the most frequent as indicated.

**Table - 4:** Characteristics of the participants (young adults aged 18-25 years) with and without obesity, including perception of satiety and personal/family medical history.

Features		Total		Without obesity		With obesity		aOR (95% CI)	p-value
		n	%	n	%	n	%		
Sex/Gender	Female	96	68.6	81	70.4	15	60.0	Reference	Reference
	Male	44	31.4	34	29.6	10	40.0	1.48 (0.6-3.7)	0.396
Race/Ethnicity	White	83	59.3	68	59.1	15	60.0	Reference	Reference
	Brown	38	27.1	30	26.1	8	32.0	1.13 (0.43-3)	0.948
	Black	19	13.6	17	14.8	2	8.0	0.56 (0.12-2.72)	0.948
Overweight or Obesity in childhood	No	96	69.6	86	76.1	10	40.0	Reference	Reference
	Yes	40	29.0	26	23.0	14	56.0	**4.45 (1.76-11.27)**	**0.003**
	Uncertain	2	1.4	1	0.9	1	4.0	7.11 (0.37-137.47)	0.194
Perception of satiety	Long satiety	78	76.5	65	82.3	13	56.5	Reference	Reference
	Short satiety	22	21.5	13	16.4	9	39.1	**3.46 (1.19-10.07)**	**0.045**
	Low satiety	2	2.0	1	1.3	1	4.4	3.51 (0,18-66.76)	0,403
Vitamin D deficiency	No	90	65.7	78	69.6	12	48.0	Reference	Reference
	Yes	47	34.3	34	30.4	13	52.0	**3.03 (1.18-7.76)**	**0.021**
Anxiety	No	85	61.6	75	66.4	10	40.0	Reference	Reference
	Yes	53	38.4	38	33.6	15	60.0	**3.94 (1.49-10.38)**	**0.005**
Depression	No	116	84.0	99	87.6	17	68.0	Reference	Reference
	Yes	22	16.0	14	12.4	8	32.0	**3.76 (1.32-10.68)**	**0.012**
Thyroid disorder	No	128	92.8	105	92.9	23	92.0	Reference	Reference
	Yes	10	7.2	8	7.1	2	8.0	1.3 (0.25-6.77)	0.756
Hypertension	No	135	97.8	112	99.1	23	92.0	Reference	Reference
	Yes	3	2.2	1	0.9	2	8.0	9.22 (0.79-107.81)	0.076
Hypercholesterolemia	No	134	97.1	110	97.35	24	96.0	Reference	Reference
	Yes	4	2.9	3	2.65	1	4.0	2.06 (0.18-23.03)	0.558
Anxiety	No	102	73.9	84	74.3	18	72.0	Reference	Reference
(family member)	Yes	36	26.1	29	25.7	7	28.0	1.12 (0.42-3)	0.822
Depression	No	111	80.4	93	82.3	18	72.0	Reference	Reference
(family member)	Yes	27	19.6	20	17.7	7	28.0	1.76 (0.64-4.83)	0.273
Thyroid disorder	No	111	80.4	93	82.3	18	72.0	Reference	Reference
(family member)	Yes	27	19.6	20	17.7	7	28.0	1.64 (0.6-4.53)	0.336
Hypertension	Yes	109	79.0	90	79.65	19	76.0	Reference	Reference
(family member)	No	29	21.0	23	20.35	6	24.0	1.02 (0.43-3.39)	0.725
Hypercholesterolemia	No	93	67.4	80	70.8	13	52.0	Reference	Reference
(family member)	Yes	45	32.6	33	29.2	12	48.0	2.29 (0.93-5.68)	0.073
Type 2 diabetes mellitus	No	80	58.0	74	65.5	6	24.0	Reference	Reference
(family member)	Yes	58	42.0	39	34.5	19	76.0	**6.38 (2.32-17.51)**	**0.00032**

aOR: adjusted Odds Ratio; CI: Confidence Interval. Adjustments were made for Sex, Race, and Age. Reference groups are the most frequent as indicated. The number of participants is variable, since some of them did not answer all the questions. The personal/family medical history was self-reported, based on previous medical diagnosis.

The comparison between the allele/genotype frequencies and obesity, overweight/obesity, and other variables was performed for all variants, since none of them showed a deviation of allele frequency from the Hardy-Weinberg equilibrium. The allele frequencies observed in the study presented values between allele frequencies of European and African populations according to the ALFA Project (ALFA: Allele Frequency Aggregator, using NCBI:dbSNP access, 2025) (Phan et al., 2025), except for *LEPR* rs1805094 variant, which presented a higher frequency of the minor allele (MAF (C): 0.230) in comparison to these populations (European MAF (C): 0.149 and African MAF (C): 0.059). However, all allele frequencies were similar to those reported by the Online Archive of Brazilian Mutations - AbraOM (Naslavsky et al., 2022). 

Considering the sample size of the 18-25 age group (n=140), the range of minor allele frequencies (MAF) observed in our population - varying from approximately 0.05 for the rarest variant (*LEP* rs17151919) to 0.37 for the most frequent (*LEP* rs7799039) - and the reported effect sizes (Odds Ratios > 2.0), the statistical power ranged from 82% to >99% ($\alpha$ = 0.05). This confirms that the sample was sufficiently powered to detect the significant associations reported, particularly for variants with moderate-to-high frequencies or large effect sizes.

**Figure 1 -:**
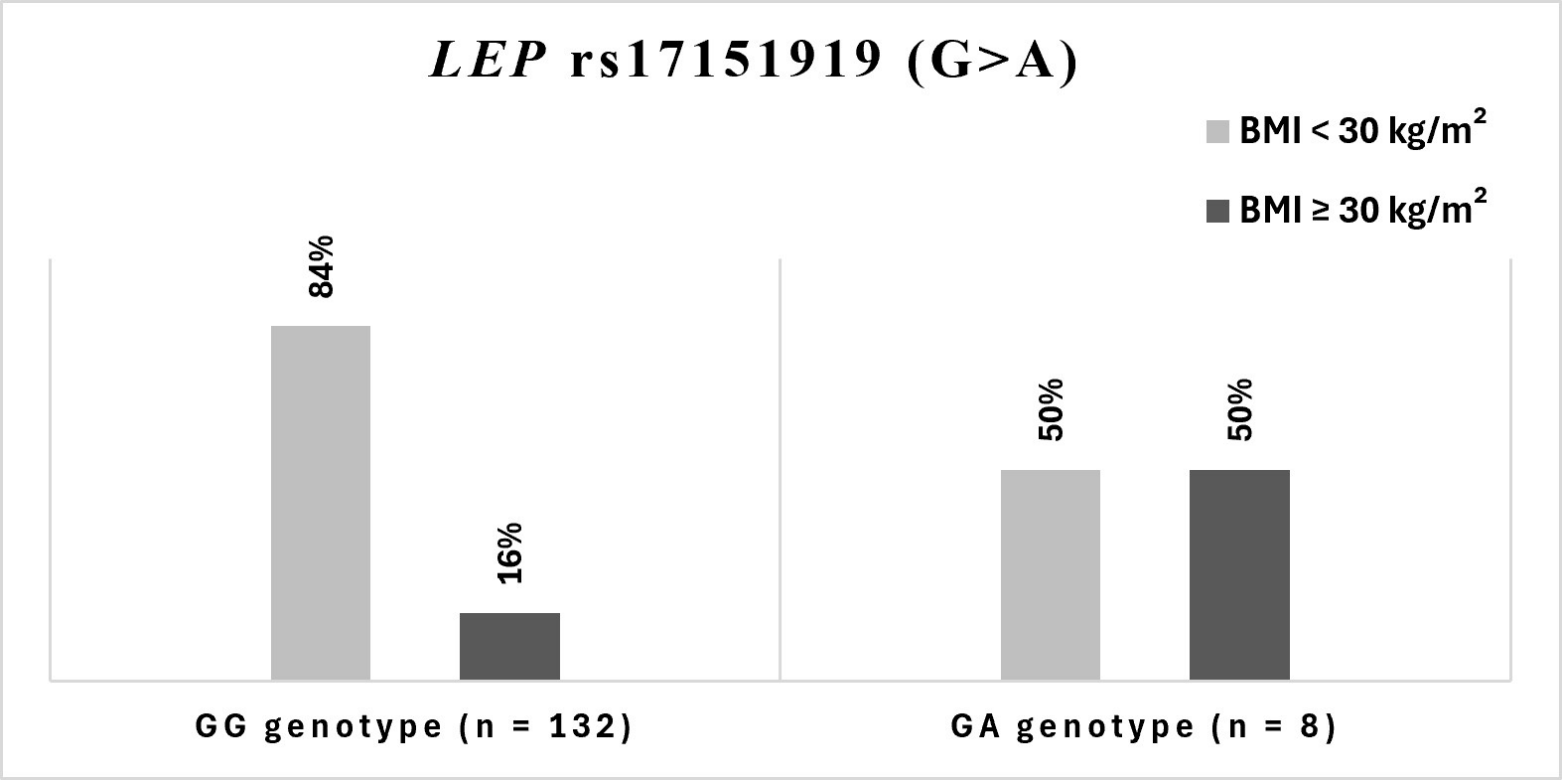
Carriers of the *LEP* rs17151919 GG and GA genotypes, with and without obesity, aged between 18 to 25 years.

The allele/genotype analysis revealed a strongly positive association between the rs17151919 variant of the *LEP* gene and obesity in young adults, showing a ninefold increased risk of individuals carrying the lower frequency allele (A) of developing obesity when compared to non-carriers (aOR: 9.02; 95% CI 1.79-45.49; p = 0.007) (Table 5). Half of the GA genotype carriers presented obesity between 18 and 25 years of age (Figure 1).

In addition, individuals carrying the alternative allele (A) of the *LEP* rs7799039 showed a 2.2-fold increased risk to present BMI ≥ 25 kg/m² (overweight or obesity) (aOR: 2.18; 95% CI 1.07-4.42; p = 0.0308), while the presence of the homozygous genotype for the alternative allele (AA) also suggests a 3.8-fold increased risk of BMI ≥ 30 kg/m² (obesity) in young adults (aOR: 3.85; 95% CI 0.95-15.54; p = 0.058) (Table 5). 

**Table 5 -:** Genotype and allele distribution of *LEP*, *LEPR*, and *FTO* variants observed in individuals aged 18-25 years, without obesity (BMI < 30 kg/m²) and with obesity (BMI ≥ 30 kg/m²).

Gene/Variant	Genotypes/ Alleles	Without obesity	With obesity	aOR (95% CI)	p-value
		n (%)	n (%)		
*LEP rs7799039*	GG	54 (46.96)	8 (32)	Reference	
(G>A)	GA	52 (45.22)	12 (48)	1.46 (0.54-3.97)	0.4599
	AA	9 (7.83)	5 (20)	3.85 (0.95-15.54)	0.0582
	nonCarrier-G	9 (7.83)	5 (20)	Reference	
	Carrier-G	106 (92.17)	20 (80)	0.32 (0.09-1.14)	0.0785
	nonCarrier-A	54 (46.96)	8 (32)	Reference	
	Carrier- A	61 (53.04)	17 (68)	1.78 (0.69-4.58)	0.2334
*LEP rs2167270*	GG	37 (32.17)	12 (48)	Reference	
(G>A)	GA	59 (51.3)	10 (40)	0.57 (0.21-1.55)	0.2743
	AA	19 (16.52)	3 (12)	0.48 (0.12-2)	0.3134
	NonCarrier- G	19 (16.52)	3 (12)	Reference	
	Carrier- G	96 (83.48)	22 (88)	1.54 (0.41-5.87)	0.524
	NonCarrier- A	37 (32.17)	12 (48)	Reference	
	Carrier- A	78 (67.83)	13 (52)	0.55 (0.22-1.4)	0.2078
*LEP rs17151914*	CC	102 (88.7)	20 (80)	Reference	
(C>T)	CT	13 (11.3)	5 (20)	1.91 (0.57-6.42)	0.2931
	TT	0 (0)	0 (0)	NC	NC
	NonCarrier- T	102 (88.7)	20 (80)	Reference	
	Carrier- T	13 (11.3)	5 (20)	1.91 (0.57-6.42)	0.2931
*LEP rs17151919*	GG	111 (96.52)	21 (84)	Reference	
(G>A)	GA	4 (3.48)	4 (16)	**9.02 (1.79-45.49)**	**0.0077**
	AA	0 (0)	0 (0)	NC	NC
	NonCarrier- A	111 (96.52)	21 (84)	Reference	
	Carrier- A	4 (3.48)	4 (16)	**9.02 (1.79-45.49)**	**0.0077**
*LEPR rs1137100*	AA	71 (61.74)	20 (80)	Reference	
(A>G)	AG	43 (37.39)	5 (20)	0.48 (0.16-1.42)	0.1846
	GG	1 (0.87)	0 (0)	NC	NC
	NonCarrier- A	1 (0.87)	0 (0)	Reference	
	Carrier- A	114 (99.13)	25 (100)	NC	NC
	NonCarrier- G	71 (61.74)	20 (80)	Reference	
	Carrier- G	44 (38.26)	5 (20)	0.46 (0.16-1.38)	0.1659
*LEPR rs1137101*	AA	31 (26.96)	8 (32)	Reference	
(A>G)	AG	66 (57.39)	15 (60)	0.98 (0.36-2.66)	0.966
	GG	18 (15.65)	2 (8)	0.56 (0.1-3.08)	0.5032
	NonCarrier- A	18 (15.65)	2 (8)	Reference	
	Carrier- A	97 (84.35)	23 (92)	1.77 (0.37-8.48)	0.4771
	NonCarrier- G	31 (26.96)	8 (32)	Reference	
	Carrier- G	84 (73.04)	17 (68)	0.9 (0.34-2.4)	0.8352
*LEPR rs1805094*	GG	72 (62.61)	15 (60)	Reference	
(G>C)	GC	36 (31.3)	9 (36)	1.15 (0.44-3.01)	0.776
	CC	7 (6.09)	1 (4)	0.43 (0.04-4.18)	0.4671
	NonCarrier- G	7 (6.09)	1 (4)	Reference	
	Carrier- G	108 (93.91)	24 (96)	2.47 (0.26-23.02)	0.428
	NonCarrier- C	72 (62.61)	15 (60)	Reference	
	Carrier- C	43 (37.39)	10 (40)	1.01 (0.4-2.59)	0.9757
*FTO rs9939609*	TT	36 (31.3)	6 (24)	Reference	
(T>A)	TA	60 (52.17)	11 (44)	0.97 (0.32-2.97)	0.9635
	AA	19 (16.52)	8 (32)	2.17 (0.62-7.57)	0.2251
	NonCarrier- T	19 (16.52)	8 (32)	Reference	
	Carrier- T	96 (83.48)	17 (68)	0.45 (0.16-1.25)	0.1272
	NonCarrier- A	36 (31.3)	6 (24)	Reference	
	Carrier- A	79 (68.7)	19 (76)	1.26 (0.45-3.55)	0.664

aOR: adjusted Odds Ratio; CI: Confidence Interval. Adjustments were made for Sex, Race, and Age. Reference groups are the homozygous genotypes of the most frequent allele (reference allele homozygotes), or non-carriers, as indicated. The underlined allele indicates the alternative allele (Minor Allele Frequency) of each variant.

In contrast, the *FTO* rs9939609 variant was not significantly associated with an increased risk of obesity or overweight in individuals 18 to 25 years of age, differing from results commonly found in *FTO* studies. 

The haplotypes analysis also revealed a very expressive result regarding the association of *LEP* variants and obesity (Table 6). The haplotype GACA of *LEP* (rs7799039 (G>A); rs2167270 (G>A); rs17151914 (C>T); rs17151919 (G>A)), which contains the alternative alleles of *LEP* rs2167270 and rs17151919, revealed a 12.7-fold increased risk of carriers of this haplotype aged between 18 and 25 years presenting a BMI above 30 kg/m² when compared to the most frequent haplotype presented by the individuals studied (aOR: 12.67; 95% CI 2.25-71.45; p = 0.004). 

**Table 6 -:** *LEP* and *LEPR* haplotypes observed in individuals aged 18-25 years, with or without obesity, and with or without overweight/obesity.

Haplotypes	Controls	Cases	aOR (95% CI)	p-value
	n	(%)	n	(%)
Obesity (BMI ≥ 30 kg/m²)				
*LEP* ^*a*^				
GACG	93 (40.43)	11 (22)	Reference	Reference
AACG	0 (0)	1 (2)	NC	NC
AGCG	70 (30.43)	21 (42)	2.51 (1.1-5.73)	0.0284
GACA	4 (1.74)	3 (6)	12.67 (2.25-71.45)	0.004
GATA	0 (0)	1 (2)	NC	NC
GGCG	50 (21.74)	9 (18)	1.58 (0.6-4.18)	0.3565
GGTG	13 (5.65)	4 (8)	2.7 (0.69-10.53)	0.152
*LEPR* ^*b*^				
AAG	82 (35.65)	20 (40)	Reference	Reference
AAC	45 (19.57)	11 (22)	0.85 (0.35-2.06)	0.7177
AGC	4 (1.74)	0 (0)	NC	NC
AGG	54 (23.48)	14 (28)	1.09 (0.49-2.43)	0.8321
GAC	1 (0.43)	0 (0)	NC	NC
GGG	44 (19.13)	5 (10)	0.51 (0.17-1.5)	0.2202
Overweight/Obesity (BMI ≥ 25 kg/m²)				
*LEP* ^*a*^
GACG	61 (42.4)	43 (31.62)	Reference	Reference
AACG	0 (0)	1 (0.74)	NC	NC
AGCG	40 (27.8)	51 (37.5)	1.93 (1.07-3.47)	0.0291
GACA	4 (2.8)	3 (2.2)	1.4 (0.28-7)	0.6834
GATA	0 (0)	1 (0.74)	NC	NC
GGCG	28 (19.4)	31 (22.8)	1.67 (0.86-3.23)	0.1269
GGTG	11 (7.6)	6 (4.4)	0.78 (0.26-2.38)	0.6675
*LEPR* ^*b*^				
AAG	53 (36.8)	49 (36.0)	Reference	Reference
AAC	25 (17.4)	31 (22.8)	1.29 (0.65-2.56)	0.4728
AGC	2 (1.4)	2 (1.5)	1.05 (0.14-7.98)	0.9629
AGG	35 (24.3)	33 (24.3)	1.01 (0.54-1.9)	0.9749
GAC	1 (0.7)	0 (0)	NC	NC
GGG	28 (19.4)	21 (15.4)	0.84 (0.42-1.7)	0.6339

aOR: adjusted Odds Ratio; CI: Confidence Interval. Adjustments were made for Sex, Race, and Age. Reference groups are the most frequent haplotypes as indicated. ^a^ Variants of *LEP* gene haplotypes: rs7799039G>A:rs2167270G>A:rs17151914C>T:rs17151919G>A; ^b^ Variants of *LEPR* gene haplotypes: rs1137100A>G:rs1137101A>G:rs1805094G>C.

Another *LEP* haplotype, AGCG (rs7799039 (G>A); rs2167270 (G>A); rs17151914 (C>T); rs17151919 (G>A)), which contains only the alternative allele of the variant rs7799039, also revealed a significant association to obesity, showing 2.5-fold increased risk of carriers of this haplotype aged 18-25 years having a BMI above 30 kg/m² (aOR: 2.5; 95% CI 1.1-5.73; p = 0.0284) and 1.9-fold increased risk of having a BMI above 25 kg/m² (aOR: 1.93; 95% CI 1.07-3.47; p = 0.0291). 

Furthermore, the following conditions were strongly associated with BMI ≥ 30 kg/m² in individuals aged 18 to 25 years old: having been a child with overweight or obesity (aOR: 4.45; 95% CI 1.76-11.27; p = 0.003); presenting short satiety (aOR: 3.46; 95% CI 1.19-10.07; p = 0.045); having vitamin D deficiency (aOR: 3.03; 95% CI 1.18-7.76; p = 0.021); self-report of having anxiety (aOR: 3.94; 95% CI 1.49-10.38; p = 0.005); self-report of having depression (aOR: 3.76; 95% CI 1.32-10.68; p = 0.012), and presenting a family history of diabetes type 2 (aOR: 6.38; 95% CI 2.32-17.51; p = 0.0003) (Table 4). According to these results, it is worth emphasizing the high odds ratios presented in all associations found.

## Discussion

Allele and genotype analyses of the *LEP* gene revealed a strongly significant association between the *LEP* rs17151919 variant and individuals aged 18 to 25 years with obesity. However, it is important to note that the 95% confidence interval for this association was wide (1.79-45.49), reflecting the low frequency of the minor A allele in our sample and indicating lower precision in the effect size estimate. Despite this limitation, the magnitude of the association is consistent with other studies that identified a link between the *LEP* rs17151919 A-allele and higher BMI in individuals aged 18 to 30 years (Friedlander et al., 2010) and significantly decreased leptin secretion *in vitro* (Yaghootkar et al., 2020). In addition, the alternative alleles of *LEP* rs2167270 and rs17151919 seem to be in linkage disequilibrium, and the combined effect of their alternative alleles present in the *LEP* GACA haplotype (rs7799039 (G>A); rs2167270 (G>A); rs17151914 (C>T); rs17151919 (G>A)) showed an even stronger association with BMI, indicating a 12.7-fold increased risk of obesity (BMI above 30 kg/m²) in the young adults studied. According to some studies, the A allele of *LEP* rs2167270 was associated with increased plasma leptin levels (Fourati et al., 2013; Duan et al., 2020), which also suggests an increased expression of the linked rs17151919 risk allele in carriers of the GACA haplotype, and consequently, an increase in its deleterious impact. 

In relation to *LEP* rs7799039 variant, the literature data presents conflicting results about the association between this variant, obesity, and related phenotypes. A study performed in Taiwan observed that carriers of this population’s minor allele (G-allele) were significantly associated with higher leptin levels in individuals with obesity and females (Duan et al., 2020). On the other hand, another study performed in Mongolian individuals with metabolic syndrome identified that carriers of AA genotype of *LEP* rs7799039 presented higher BMI when compared to GA and GG genotype carriers (BMI ≥ 36.5 ± 2.49) (Dagdan et al., 2018), corroborating our findings that showed a 2.2-fold increased risk of overweight or obesity associated to carriers of the A-allele and suggested a 3.8-fold increased risk of obesity associated to carriers of AA genotype. Moreover, an *in vitro* study showed that carriers of genotype AA presented leptin mRNA levels 60% higher in adipose tissue cells than GA/GG, and that mRNA levels remained higher even after adjusting for BMI. In addition, serum leptin levels were approximately 50% higher in AA than in GA/GG genotype carriers (Hoffstedt et al., 2002). Thus, based on these findings, the A-allele suggests increasing leptin levels, with a potentiated effect on homozygous carriers, which could lead to earlier hyperleptinemia and leptin resistance in young adults. This condition could reduce the feeling of satiety and increase food consumption in a differentiated way in individuals with the genotype of risk. 

In respect to contradictory findings in Taiwan population (Duan et al., 2020), it is important to note that, unlike the Brazilian population, the G-allele of *LEP* rs7799039 is the allele of minor frequency in Taiwanese population (MAF (G): 0.276), while the A-allele is the allele of minor frequency in the European (MAF (A): 0.348), African (MAF (A): 0.037), and Brazilian populations (MAF (A): 0.368) (Naslavsky et al., 2022; Phan et al., 2025). Therefore, the very distinct allele frequency of *LEP* rs7799039 and the different genetic profiles would probably explain the distinct effects of this genetic variant on obesity development observed among the different populations. In this context, a study performed in Brazilians from different regions of the country analyzed autosomal biparental markers revealing very elevated levels of genetic admixture between European, Amerindian, and African, with predominant European ancestry, in all regions studied (Pena et al., 2020). Therefore, it is important to emphasize that Brazilians present a heterogeneous ancestry, which results in genetic variant frequencies commonly closer to those observed in Europeans, Amerindian, and Africans than in other populations.

Corroborating the association of *LEP* rs7799039 A-allele and higher BMI, the haplotype analyses of individuals aged 18 to 25 years with obesity also revealed that carriers of the *LEP* AGCG haplotype, which contains the alternative A-allele of *LEP* rs7799039, show a 2.5-fold increased risk of obesity and a 1.9-fold increased risk of being an individual with overweight or obesity. However, the alternative allele of this variant seems to be in linkage disequilibrium with the reference G-allele of *LEP* rs2167270, which makes it impossible to perform a combined analysis of its effects, where both alternative alleles are present. Therefore, the results of the present study suggest that the A-allele of *LEP* rs7799039 has a dominant effect on overweight/obesity expression and an additive effect on obesity expression in young adults. Nevertheless, in view of the divergences found in literature, further studies are needed to clarify the role of this variant in weight gain, its interaction with variants of other genes, and its differentiated effect in individuals of different age groups. 

Regarding the lack of association between *LEPR* variants and obesity in young adults, our finding aligns with a study performed in Australian women that evaluated longitudinal changes in body composition. The study revealed a small effect on body mass, and that the effects of *LEPR* variants on adiposity only become apparent after several years (de Silva et al., 2001). These findings suggest that the impact of *LEPR* variants is proportionally lower than other genetic risk factors for the development of obesity, presenting a significant contribution to this phenotype only in the long term.

Interestingly, the *FTO* rs9939609 variant showed no association with obesity in young adults, corroborating a study with Brazilian adolescents aged 18 to 19 years (Rodrigues et al., 2020).

At the transcriptional level, Berulava and Horsthemke (2010) observed that even in heterozygous individuals, the expression of the *FTO* rs9939609 alternative allele (A) is higher than the expression of the ancestral allele (T), which corroborates the effect of the risk allele on obesity. Previously, Fischer et al. (2009) had reported that the inactivation of the *FTO* gene expresses a protective effect against obesity, revealing that the increased levels of *FTO* expression, such as that observed in carriers of the *FTO* rs9939609 A-allele, would be the cause of this association with obesity phenotype. 

Data from the literature show that the *FTO* rs9939609 and even other variants located in *FTO* intronic regions may be associated with obesity due to their interaction with the promoter region of the *IRX3* gene, which is expressed in hypothalamic pro-opiomelanocortin neurons (POMC). Changes in its expression levels affect body adiposity, and energy expenditure that is associated with the regulation of body mass. These findings may explain the strong association of variants in the intron of *FTO* with obesity (Smemo et al., 2014; Schneeberger, 2019). 

Therefore, a possible explanation for the differentiated impact of *LEP* and *FTO* variants in individuals from different age groups could be the distinct metabolic pathways involved in the pathogenesis of obesity, suggesting an earlier effect of *LEP* rs17151919 probably due to the greater impact of lower levels of leptin specifically on early-onset obesity. In contrast, a possible later effect of *FTO* rs9939609 suggests an initial association with changes in the regulation of body mass, favoring adiposity, and subsequently leading to increased risk for hyperleptinemia, leptin resistance, and obesity. However, in either case, the effects of genetic risk factors tend to be progressively enhanced throughout life due to the action of additional risk factors, such as sedentary lifestyle, stress and diet rich in foods with high energy density and low nutritional value, resulting in greater impacts on the development of obesity later in life.

Concerning some limitations of the study, the reliance on self-reported anthropometric data for a portion of the sample, while validated against a subgroup, may introduce recall bias. Furthermore, a larger number of participants and their serum leptin data would certainly improve the chances of identifying variants with smaller impacts and contribute to better understanding of the variant’s effects. 

Lastly, this study also showed that young adults present significant association between higher risk of obesity and the perception of short satiety, anxiety, depression, vitamin D deficiency and family history of type 2 diabetes, emphasizing important risk factors for early-onset obesity and suggesting more careful medical and nutritional monitoring in these cases.

In conclusion, this study identified important biomarkers for predicting the higher risk of developing overweight and obesity in young adults, suggesting a differentiated impact of these genetic variants in this age group. The twofold increased risk of developing overweight and obesity associated with the alternative allele of the *LEP* rs7799039 variant underscores the potential utility of this variant as a predictive biomarker for weight gain in young adults, particularly given its high frequency in the study population and others globally. Despite the lower frequency of the *LEP* rs17151919 minor allele (ranging from 1% to 10% in different populations, according to ALFA project), the ninefold increased risk of obesity associated with the alternative allele of this variant also suggests its high relevance for the prediction of early-onset obesity. Therefore, the risk factors associated with the expression of overweight and obesity identified in this study contribute to broadening our understanding of this complex condition, in addition to highlighting important genetic tools for obesity prevention, and treatment strategies based on precision medicine and personalized nutrition.

## Data Availability

The entire dataset supporting the results of this study was published in the article itself.
